# The Role of Social Network Technologies in Online Health Promotion: A Narrative Review of Theoretical and Empirical Factors Influencing Intervention Effectiveness

**DOI:** 10.2196/jmir.3662

**Published:** 2015-06-11

**Authors:** Panos Balatsoukas, Catriona M Kennedy, Iain Buchan, John Powell, John Ainsworth

**Affiliations:** ^1^ Institute of Population Health Centre for Health Informatics University of Manchester Manchester United Kingdom; ^2^ Health eResearch Centre Farr Institute for Health Informatics Research University of Manchester Manchester United Kingdom; ^3^ School of Computer Science University of Birmingham Birmingham United Kingdom; ^4^ School of Computer Science University of Manchester Manchester United Kingdom; ^5^ Nuffield Department of Primary Care Medical Sciences Division University of Oxford Oxford United Kingdom

**Keywords:** health behaviors, health promotion, health behavior change, health education, social media, social technology, social networking, content analysis, theoretical grounding

## Abstract

**Background:**

Social network technologies have become part of health education and wider health promotion—either by design or happenstance. Social support, peer pressure, and information sharing in online communities may affect health behaviors. If there are positive and sustained effects, then social network technologies could increase the effectiveness and efficiency of many public health campaigns. Social media alone, however, may be insufficient to promote health. Furthermore, there may be unintended and potentially harmful consequences of inaccurate or misleading health information. Given these uncertainties, there is a need to understand and synthesize the evidence base for the use of online social networking as part of health promoting interventions to inform future research and practice.

**Objective:**

Our aim was to review the research on the integration of expert-led health promotion interventions with online social networking in order to determine the extent to which the complementary benefits of each are understood and used. We asked, in particular, (1) How is effectiveness being measured and what are the specific problems in effecting health behavior change?, and (2) To what extent is the designated role of social networking grounded in theory?

**Methods:**

The narrative synthesis approach to literature review was used to analyze the existing evidence. We searched the indexed scientific literature using keywords associated with health promotion and social networking. The papers included were only those making substantial study of both social networking and health promotion—either reporting the results of the intervention or detailing evidence-based plans. General papers about social networking and health were not included.

**Results:**

The search identified 162 potentially relevant documents after review of titles and abstracts. Of these, 42 satisfied the inclusion criteria after full-text review. Six studies described randomized controlled trials (RCTs) evaluating the effectiveness of online social networking within health promotion interventions. Most of the trials investigated the value of a “social networking condition” in general and did not identify specific features that might play a role in effectiveness. Issues about the usability and level of uptake of interventions were more common among pilot studies, while observational studies showed positive evidence about the role of social support. A total of 20 papers showed the use of theory in the design of interventions, but authors evaluated effectiveness in only 10 papers.

**Conclusions:**

More research is needed in this area to understand the actual effect of social network technologies on health promotion. More RCTs of greater length need to be conducted taking into account contextual factors such as patient characteristics and types of a social network technology. Also, more evidence is needed regarding the actual usability of online social networking and how different interface design elements may help or hinder behavior change and engagement. Moreover, it is crucial to investigate further the effect of theory on the effectiveness of this type of technology for health promotion. Research is needed linking theoretical grounding with observation and analysis of health promotion in online networks.

## Introduction

### Background

Social networking sites (SNS)—such as YouTube, Facebook, and Twitter—have been used extensively in public health and prevention interventions to change behavior and improve health outcomes [1,2]. Several aspects of SNS—including social support, empowerment, peer pressure, and interactive information-emotion sharing—have the potential to influence patients’ health behaviors and increase adherence to and engagement with such interventions [3-5]. Yet little is known about the actual effect of SNS on behavior change and on the factors that may influence user interaction and experience, such as usability, user satisfaction, and level of technology acceptance or engagement. Therefore, there is a need to understand the effectiveness of SNS in the context of wider health promotion methods and evidence—not simply assuming that interventions can be ported from one medium to another.

Previous reviews of the literature have provided mixed results about the effectiveness of SNS for health promotion with many authors characterizing the effect of online social networking on behavior change as positive, but not statistically significant [1,6]. For example, Korda and Itani [7] identified both positive and less successful examples of the application of social media (including blogs, forums, video-sharing, and wikis) for health promotion. However, the authors also concluded that there is a need for precise evaluation metrics and for behavior change interventions to be grounded in theory in order to successfully measure and assess their effectiveness. The previous work in this area suggests that the lack of clear evidence can be attributed to the following factors.

First, there are a small number of randomized controlled trials (RCTs) with considerable heterogeneity used to evaluate the actual effect of online social networking on behavior change. This was evident in two recent systematic reviews, with a meta-analysis, by Maher and Lewis [1] and Lavanjo et al [2], which showed mixed results. Maher and Lewis showed a modest effect for the examined interventions on behavior change when magnitudes of the effect sizes were calculated, while Lavanjo et al reported a slight positive effect of SNS interventions on health behavior-related outcomes. However, the findings of these two studies should be interpreted with caution since, in the case of both reviews, the authors analyzed a small number of RCTs (six studies in [1] and eight in [2]), the majority of which were short-term trials, with a study duration not exceeding 6 months, while there was considerable heterogeneity of study designs, evaluation metrics, health topics, and types of SNS.

Further, there is a lack of ecological validity due to the difficulty in assessing the true effect of SNS in the context of multi-component interventions. There is a lack of clarity over whether a positive effect could be attributed to the SNS or the non-SNS component of an intervention [1,2,8]. A typical example of this phenomenon was highlighted by Chang et al [9] who reviewed the evidence about the effect of SNS on weight management behaviors. From the 20 studies that met the eligibility criteria for this review, only one study measured the “isolated effect” of social media. The authors cautioned that in the case of the remaining studies it was difficult to assess whether a reported effect was related to a social media component alone or was a synergistic effect. This problem was also reported in other reviews of social media use in behavior change and health promotion, such as Schein et al [10] who reviewed the effectiveness of social media in public health communication, or the review by Hamm et al [6] who were focused on the behaviors of patients and caregivers.

There is also a lack of knowledge about the role of theory in the effectiveness of SNS-enabled interventions. Although studies have shown a positive effect of theory-driven Internet-based interventions on behavior change [11], there is little evidence in the context of SNS [1,2]. Understanding this phenomenon is important for the design of interventions. Yet, more research is needed to review existing evidence in this context and identify the type of theories and models currently used in the delivery of interventions through SNS, but also for the design of the social networking application itself.

Finally, previous literature reviews in the area of SNS for health promotion have focused on summative and outcome evaluations rather than formative and process assessments. For example, most reviewers in this field have attempted to examine the effect of SNS on objectively measured behavior change usually though the use of RCTs or some form of experimental study, like pre-test and post-test evaluations [1,2]. However, other factors that may have an important influence on the effectiveness of SNS, such as usability, user satisfaction, and level of technology acceptance or engagement, have rarely been synthesized. While these types of evaluation cannot provide direct evidence on effectiveness, they may provide very useful insights to guide future intervention development and implementation. For example, usability factors may influence which features of the delivered intervention are actually used, thus limiting its actual effectiveness. This type of information is usually included in research and technical papers reporting work in progress or complete research documenting the results of an iterative evaluation process. To date, a significant number of this type of studies has not met the eligibility criteria for inclusion in traditional RCT-focused systematic reviews.

Therefore, the aim and originality of this current review is to extend our knowledge about the effectiveness of SNS for health promotion by addressing some of these gaps in the existing literature, in particular, (1) extending the focus on effectiveness by reviewing studies reporting findings relevant to the usability, user satisfaction, acceptance, and level of engagement with SNS, as well as studies using different research methods and techniques, beyond traditional RCTs, to evaluate effectiveness, such as observational, qualitative, and pilot studies; (2) focusing on studies and findings that apply directly to the isolated effect of SNS (wherever this is possible); and (3) to investigate the extent to which theory has contributed to the design of SNS-driven interventions.

This paper is structured as follows. First, we present definitions of concepts that are central to this review. The next section presents the methods used to review the literature as well as the decisions made to select studies for review. In the following section, we present the findings of this review, while the final section includes a discussion and some conclusions.

### Definitions

Our use of the term “social networking sites” (SNS) or “social networking” includes the broader concepts of Health 2.0 and Medicine 2.0. The definitions of these concepts have been previously reviewed [12]. They identify the two most important features as (1) patient/consumer participation and (2) Web 2.0 technology (user-generated content). There are several examples of different types of SNS that have been used for health promotion. For example, YouTube has been frequently used for the promotion of information about cancer screening, as well as obesity and dietary problems [13,14], Facebook has been used in interventions related to sexual health issues [15], and Twitter has been incorporated in the design of interventions about prenatal health promotion and education [16]. In addition to publicly available popular SNS (like Facebook), there is also a considerable number of standalone health-focused social networking applications used for conditions like obesity [17], healthy living [18], as well as various chronic diseases, like diabetes [19].

In the context of this review, the term “health promotion” is used in a broad sense to include health education initiatives (eg, in schools), social marketing campaigns (eg, using advertising), community development, and behavior change interventions (eg, smoking cessation websites). It can also take the form of educators in social networks to direct non-experts towards relevant and accurate health information. Agents with this role (which may be people or tools) have been called “apomediaries” [20]. Examples include knowledgeable collaborative filtering and recommendation agents. Despite the fact that health promotion is not synonymous with health prevention strategies, like social marketing and health education, in the context of our study, health promotion is used as an umbrella term to include also interventions grounded in social marketing and health education approaches. This decision was made because to date there are several successful examples of integrative health promotion interventions using social marketing methods and approaches, like audience segmentation [21,22], or health promotion interventions applying health education strategies to promote behavior change [23].

In this paper, we consider studies of “effectiveness” to encompass evaluation of measured behavior change (eg, RCTs and controlled studies), as well as aspects of the user experience and interaction with the SNS application that might help or hinder behavior change, such as usability, user satisfaction, technology acceptance, and level of engagement. “Usability” refers to the ease of use of the SNS application and is normally measured using behavioral metrics, like effectiveness, efficiency, learnability, and errors [24]. “User satisfaction” reports on the subjective satisfaction with the interface components of a given application [25]. “User engagement/adoption” includes the reporting of statistical figures about the level of adherence with a given intervention. This information may be reported both in terms of participation rate in the online intervention, but also in terms of Google analytics indicators, like number of hits or posts, and time spent. Finally, the term “technology acceptance” is used in a broad manner to include both the level of uptake of a given technology, but also more formal studies focused on modeling factors influencing user acceptance of technology, such as the Technology Acceptance Model [26].

Expectations about social networking, such as motivational support and peer-pressure, may be grounded in social or behavioral theories. For example, the Theory of Planned Behavior [27] predicts that norms of significant people in an individual’s social circles (subjective norms) have a strong influence on the individual’s behavioral intentions. Similarly, Social Cognitive Theory [28] predicts social learning by observation, which can take place in social networks. In the context of this review, the term “theory” is used broadly to include any theory used as the basis for the design of an intervention delivered through online social networking. In the absence of specific theory, we examined for the presence of a specific model or technological approach used to inform the design and delivery of interventions through SNS.

## Methods

### Overview

The narrative synthesis approach to literature review was used to analyze the existing evidence. This decision was made because the aim of this review was to synthesize evidence from a heterogeneous body of literature with studies representing different health promotion initiatives with a range of effectiveness evaluation measures and mixed-method research designs [29].

As guidance to this review, we followed the method of narrative synthesis prescribed by Rodgers et al [29]. Key elements of this method were (1) the development of a preliminary synthesis, and (2) the exploration of relationships (differences and similarities) within and between homogeneous groups of studies. For the development of a preliminary synthesis, we used two techniques: (1) tabulation, as a means of extracting and organizing data from the primary studies in tables, and (2) grouping/clustering, which involved an interpretivist analysis of the contents of the primary studies in order to identify dominant groups of studies that shared a common set of characteristics. More details about the preliminary synthesis are presented in the following subsections. After the preliminary synthesis, the data collected were used to explore relationships between primary studies both at the individual and group level.

### Scoping Search and Searching Process

We undertook an initial scoping search of the literature using Google Scholar. The purpose of this initial search was to gain a feel about the important aspects of the topic of this review, and more specifically to identify the different types of SNS available and to explore different areas of health promotion where SNS can play an important role. The results of the initial scoping review informed the design of our search strategy.

We searched Google Scholar and PubMed using a search strategy conceptualized as the following: Health AND “behavior change” AND <health promotion keywords> AND <social networking technology keywords>. The full search terms were health AND “behavior change” AND (“health promotion” OR “health education” OR “social marketing” OR “intervention” OR “persuasive” OR “therapy”) AND (“social networking” OR “social media” OR “peer-to-peer” OR “online forum” OR “online community” OR “virtual community OR “online discussion” OR “electronic support groups” OR “participatory” OR “citizen-led” OR “web 2.0” OR “medicine 2.0” OR “user-generated content” OR “social software” OR “collaborative software”).

The identification of a broad range of studies was one of the main challenges of this review. For this purpose, we decided to search using the Google Scholar (in addition to the PubMed database). Empirical studies [30,31] have shown that Google Scholar provides sufficient coverage to be used reliably in literature reviews of this kind. The date range was January 2005 to December 2013. Only articles written in English were included. Keyword searches were conducted in January 2014.

### Inclusion/Exclusion Criteria

We included articles on health promotion (HP) interventions, where online SNS was a major theme in the study. In particular, these included the following: (1) Evaluation of interventions combining HP with SNS, including studies of effectiveness in terms of behavior change, usability, user satisfaction, level of engagement, and technology acceptance; (2) Observational studies of a social network within an existing HP intervention, including those involving content analysis, social network analysis or other usage patterns, but excluding studies of general social networks where health was one topic, unless the discussions were connected to an HP initiative; and (3) Designs and planned interventions were included if they addressed the relationship between HP and the anticipated emergent features of SNS. We also included papers reporting planned methodologies for the evaluation of interventions, as well as papers reporting work in progress, such as evaluation of early prototype designs. Information extracted from these papers contributed to our understanding of the different methods available for the evaluation of the effectiveness of interventions, and the presence of theories as evidence for guiding the design of interventions with an HP and an SNS component.

The following were excluded: mention of social networking in a generic, non-specific way; use of a discussion board as an “added extra” in an intervention without any significant role in the study; use of the term “social networking” to indicate “top-down” dissemination only (eg, using mobile phones or text messaging) without mention of peer-to-peer communication or other emergent SNS effects; study of health discussions on general social networks in which there is no HP initiative; and discussion/position papers, including definitions and research roadmaps (but some are cited as background).

### Data Extraction and Synthesis Process

Two of the authors (PB and CK) performed the review working independently. They extracted data on effectiveness (broadly defined) and theoretical grounding. The items extracted are shown in Multimedia Appendix 1. Disagreements during the study selection and data extraction process were solved after consultation with the other authors (IB, JA, and JP).

We did not use a specific quality assessment tool due to the heterogeneity of study designs and the varying level of completeness of the studies included in this review. However, we did make individual assessments of the internal validity of the studies. In the results, we present the research design used by each selected study and the nature of the findings reported in the individual studies, including objectively and subjectively reported measures; long-term and short-term designs; strong and weak associations, or no associations (for observational studies); positive, negative, or mixed results (in the case of pilot and qualitative studies); and significant/not significant findings (for RCTs and controlled studies) (a detailed description is provided in Multimedia Appendix 1). This information was assessed during the tabulation process. Finally, we performed an interpretivist analysis to categorize primary studies into groups and examine the relationship between them.

## Results

### Overview

**Figure 1:**
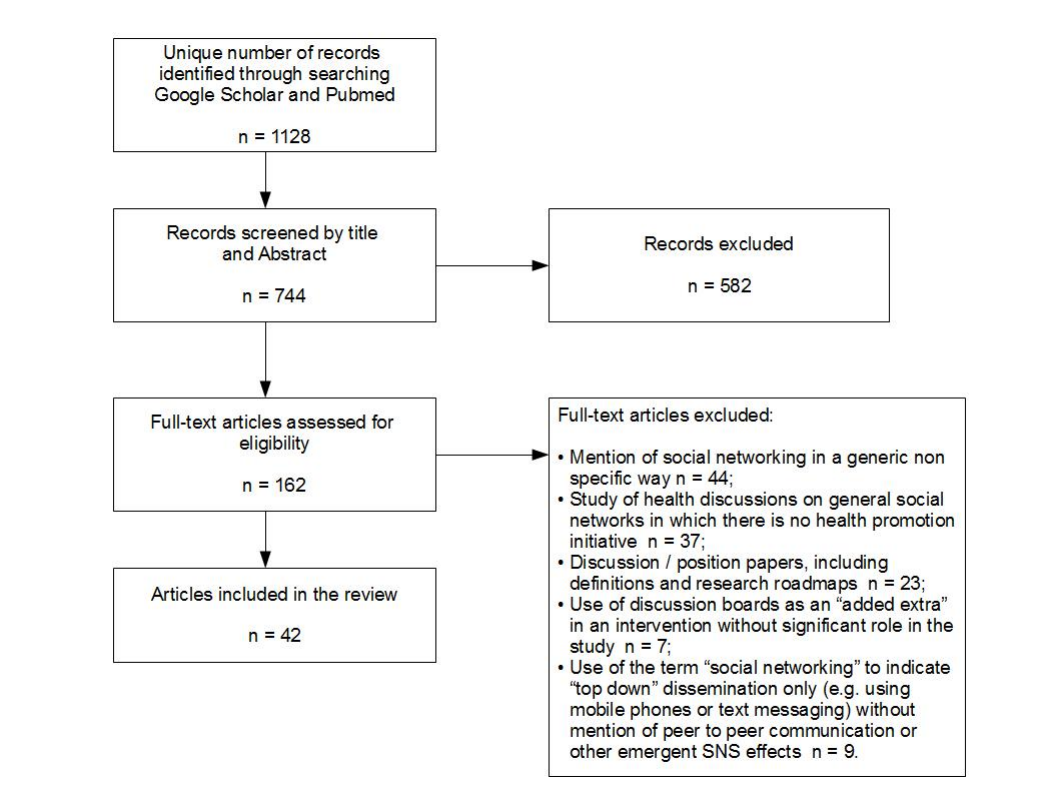
Flow of studies through the review.

The search identified 162 potentially relevant documents after review of titles and abstracts. Of these, 42 satisfied the inclusion criteria after full-text review (Figure 1). Results on effectiveness, with details about the type of study design and main findings are shown in Table 1. The use of theory in interventions, as well as the extent of top-down, theory-based approaches, and bottom-up participation (observation) is shown in Table 2.

**Table 1:** Effectiveness evaluation (summary of study types and findings).

Reference/ project or intervention name	Health topic/ Study population	Social networking topic/key words/ technology	Type of study/methods	Main findings	Effectiveness evaluations (if any)^a^
An et al, 2008 [32] (Quitplan)	Smoking cessation/adults	Active and passive online community participation	Observational study: Bi, multivariate, and path analysis to determine association between online activities and abstinence	Weak association between active community engagement and abstinence	SNS; Abstinence: +
Baghaei et al, 2009 [33] (SOFA)	Obesity/families	Motivational support; involve families	Pilot trial: will users engage with educational content? What kind of profile increases engagement?	Educational content attracted positive attention; individual profiles better than whole family	SNS+HP; Acceptance: +
Burke & Oomen-Early, 2008 [34]	General/ High School students	Blogging; community debates; advocacy campaigns	Education idea	N/A (concept only)	N/A
Cobb et al, 2010 [35] (QuitNet)	Smoking cessation/ QuitNet users	Online social support	Social network analysis: determine SNS effects (persistence, peer-to-peer communication, heterogeneity); compare with other SNS; characterize participants and subgroups	SNS effects are present; most integrated are female and older	N/A
Cunningham et al, 2008^b^ [36] (Alcohol_HelpCenter)	Problem drinkers	Online social support	Usage patterns and message content analysis: determine quality of interactions	Qualitative: content appears valuable and supportive	SNS+HP; Acceptance: +
Falan et al, 2011 [37] (SCEDES)	Diabetics	Community support and education	Concept: minimize hospitalizations	N/A	N/A
Foster et al, 2010 [38] (StepMatron)	PA/ office workers	Social influence: competitive step-counting (FaceBook app)	Pilot trial:10 nurses, 9F, 1M	9/10 walked more in social condition than in non-social (Stat. significance tested)	SNS; Objectively measured behavior change (walking): +
Fukuoka et al, 2011 [39]	Diabetes prevention/ overweight, sedentary adults	Mobile peer to peer support	Qualitative focus- group analysis to determine desired features of planned mobile intervention	Real-time peer support emerged as desirable (also, tailored advice, self-monitoring)	N/A
Gasca et al, 2009 [17] (pHealthNet)	Obesity/ adults with weight-related health problems	Persuasive and SNS technology for existing support-groups (pedometer, Web portal, mobile app)	Field study of support groups: low sustainability of behavior changes; technology evaluation: 12 patients: compare behavior during and after technology-assisted group sessions (2 subgroups of 6)	Semi-quantitative: sustained PA changes 2 wks after technology-enabled session (3 wks). Positive acceptance of technology	SNS+HP; Observational study weak association (low sustainability of behavior change): +; Acceptance: +
Gay et al, 2011 [40] (AURORA)	Emotional awareness/ adults	Mobile sharing of emotions (Web and mobile app)	Pilot study, 65 adults, 7 days. Random (EMA) assessments and post-study survey	EMA and post-study results positive for emotion awareness, sharing and social support (also among strangers), but danger of negative contagion	SNS; Emotional health: + contagion danger: -
Kamal et al, 2010 [41]	Nutrition/ general	Theory-based social networking software	Prototype development	N/A	N/A
Kharrazi et al, 2011 [42]	Obesity/ general	Online sharing of progress and peer-pressure (Facebook app)	Technology design	N/A	N/A
Krukowski et al, 2008 [43] (VTrim)	Obesity/ adults	Weight loss websites with online social support as a feature.	Observational study: Determine what elements of a website (VTrim) are associated with actual weight loss. Exploratory factor analysis; 123 overweight adults; 1 yr: treatment: months 0-6; maintenance months 7-12	In maintenance phase, “social support” was best predictor for additional weight loss. “Feedback” was best predictor during initial phase	SNS; Weight loss maintenance: +++
Lindsay et al, 2009 [44]	Exercise, smoking, diet/ coronary heart patients in deprived urban area	Online support community	RCT: determine effects of removing moderator support from online community: 108 participants, 12 months, non-moderated phase after 6 months; randomly assign half to Web-portal access and half to non-Web portal group	Significant reduction in self-reported health behaviors 3 months after moderator withdrawal (for both groups); during moderated phase, Web portal access led to positive behavior changes	HP; Self-reported health behavior: +++
Linehan et al, 2010 [45] (Tagliatelle)	Obesity/ adults	Social photo tagging of meals for nutritional content	Pilot usage and acceptability study: 14 participants	9/14 participants regularly used system over 7-day trial	SNS; Acceptance: +
Liu & Chan, 2010 [46]	General health	Seeking help in virtual communities	Research design: determine relation between social identity, beliefs, and help-seeking behavior (planned survey)	N/A	N/A
Maibach et al, 2007 [47]	General health	Social networks as ecological fields of influence	Conceptual framework for social marketing to mobilize health-promoting dynamics in social networks	N/A	N/A
Munson et al, 2010 (3GT) [48]	Positive psychology/ adults	Facebook app (3GT) for sharing positive experiences (“good things”)	Survey of 3GT users (190 participants) to record usage patterns and attitudes	Positive acceptance of app, but concern about privacy; indifference about reminders	SNS+HP; Acceptance: +/ -
Nahm et al, 2009 [49] (TSW)	Hip fracture prevention/ older adults	Educational discussion board	Exploratory qualitative analysis (316 forum posts; 245 participants)	Emergent themes included sharing of health behaviors, problems, and opportunities; also social support	N/A
Nordfelt et al, 2010 [19] (Diabit)	Diabetes/ children and parents	Peer-to-peer chat and blogging on a Web 2.0 portal	Qualitative content analysis of essays written by portal users (19 parents, 5 young people 11-18 years)	Message boards and chats found to provide valuable information that could not be provided by clinicians (attitudes to website itself were mixed)	N/A
O’Grady et al, 2008 [50]	General health	SNS for collaborative learning	Proposal of Experiential Health Information Processing Model	N/A	N/A
Olsen & Kraft, 2009 [51]	General health	SNS role in providing social support and adherence	Pilot study to determine which aspects of SNS are important in social support and adherence (semi-structured interviews, 5 participants, qualitative analysis)	Social support provided mostly by close friends or family; adherence may be improved with dynamic and interactive features (eg, games, contests)	N/A
Potente et al, 2011 [52]	Sun protection/ Australian youth	Social Media Marketing (SMM)	Online survey and thematic analysis of comments to determine effects of an SMM music video on attitudes and risk-awareness	Positive stat. significant difference in attitudes between video-exposed respondents and non-video-exposed	SNS+HP; Self-reported risk-awareness: ++
Rhodes et al, 2010 (CyBER/ M4M) [53]	Human immunodeficiency virus (HIV) prevention/ men who have sex with men (MSM)	Educators in Internet chat rooms	Quantitative analysis of participant survey (n=210); qualitative analysis of chat content (n=1851): private and public messages	Inconsistent condom use: 27% (77% of HIV positive chatters): Qualitative: need for prevention information; privacy, and trust important; educators had to respect culture	N/A
Richardson et al, 2010 [54] (Stepping Up to Health - SUH)	PA/ adults	Online community in Stepping Up to Health website	RCT: effect of online community in website. n=324; (5:1 randomization, larger number in community condition); Objective measures: pedometer data, community usage (activity) and intervention completion rates	Online community more engaged and more likely to complete intervention than non-community; otherwise no great difference in walking. However, within online community, active participants (with more posts and page views) walked more than less active participants	SNS; Adherence: ++++
Roblin, 2011 [55]	Diabetes/ patients and families	Mobile peer support for glucose management	Pilot study: experience of patients and their peer supporters using mobile technology for encouraging and reminding	Self-reported improved self-monitoring and encouragement through mobile communication with peer-supporter	SNS+HP; Acceptance: +
Stoddard et al, 2008 [56] (Smokefree.gov)	Smoking cessation/ adults	Bulletin board in website	RCT: effect of bulletin board (BB) in website. n=1375 (50:50 allocation BB vs usual)	In BB condition, only 11% posted or viewed messages; no significant difference in cessation; more time on website for BB condition; no difference in satisfaction	SNS; Abstinence: 0
Toscos et al, 2010^b^ [57]	Barriers to Physical activity/female forum users of *GetFit!*	Online forum on PA	Qualitative Analysis of *GetFit!* Forum content; compare with literature survey on barriers.	Differences between PA barriers emerging in forums and those from surveys; *GetFit!* intervention not aware of them	N/A
Waters et al, 2011 [58]	Student health	Facebook profiles of University Health Centers	Content analysis to determine the extent of “dialogic principles” (eg, usability, conversation of visitors, feedback options)	Least applied dialogic principles were feedback options (contact details) and promoting return visits. Significant relation between social networking extent (friends, fans) and use of dialogic principles	N/A
West et al, 2011 [59]	Breastfeeding	Blogging	Determine extent of blogging to support breastfeeding behavior: qualitative and quantitative analysis of posts and comments; 32 active blogs, 354 posts, 881 comments	Reports on one’s own behavior and personal experience sharing were more likely to elicit behavioral intention than advice or information. Attitude (like/dislike) most common theme in blog posts (28%); praise (support) for breastfeeding most frequent comment (43%)	SNS; Behavioral intention: ++
Woodruff et al, 2007 [60]	Smoking/ adolescents	Virtual chat room	RCT: determine effect of intervention with MI and virtual chat room (n=136)	Short-term: self reported smoking reduction for intervention group; long-term: not significant	SNS+HP; Self-reported behavior: ++
Young et al, 2010 [61]	PA/ teenage girls	Micro-blogging	Pilot study: 4 students; determine if peer-pressure and SNS technology can influence girls to exercise	Positive behavior change, gradual increase in number of steps over 4 weeks	SNS; Behavior change: +
Kamal et al, 2013 [18]	Healthy living / Adults	VivoSpace	Pilot study: interviews, questionnaires, and prototyping. Aim was evaluation in terms of usability of a novel theoretical framework (Appeal, Belonging, Commitment) for design of a social networking tool for healthy living	Findings showed ABC framework in combination with iterative usability evaluation to be promising for user engagement; but, since the study was focused on prototypes and not fully working systems, no tangible data on actual nature of engagement and its effect on health behavior change	SNS +HP; Engagement: +
Baelden et al, 2012 [62]	Acquired Immune Deficiency Syndrome (AIDS) and HIV/ Adults	Online discussion group	Pilot study: examining suitability of an anonymized discussion forum for increasing interpersonal communication and engagement in the area of HIV / evaluation through usage statistics & focus group interviews	Mixed on suitability of online discussion forums for interpersonal communication about AIDS. Use of discussion forum was successful when integrated into the curriculum. Usage was lower when participants had to use the forum on a voluntary basis	SNS; Adherence and technology engagement: +/-
Ploderer et al, 2013 [63]	Smoking cessation / Adults	Facebook support group	Pilot study: Examining the relationship between stage of health identity change and seek for social support / thematic analysis of messages posted in a public Facebook support group	Findings showed that supportive responses and leadership came from users who just started their behavior change process rather than people who had successfully completed it	SNS + HP; Self-reported behavior change: ++
Gold et al, 2012 [64]	Sexual health / Young people	Facebook + YouTube	Pilot study: Review of challenges related to promotion of sexual health behavior through Web 2.0 / usage statistics, satisfaction questionnaires, and focus groups	Mixed results in terms of adherence and engagement with technology	SNS; Adherence or technology engagement: +/-
Nguyen et al, 2013 [65]	Sexual health / Young adults	Facebook + SNS	Pilot study: Review of challenges related to promotion of sexual health behavior through Web 2.0 / usage statistics and questionnaires	Mixed results on effectiveness. The project reached 900 fans across 5 Facebook pages. Key challenges included a lack of viral recruitment, evoking substantial interest, and maintaining user engagement	SNS; Adherence or technology engagement: -
Kolt et al, 2013 [66]	Physical activity	Walk 2.0 project (blogs, social networking, virtual walking groups, forums)	RCT: A methodology to compare the effectiveness between Web 1.0, Web 2.0 and control interventions) using larger sample size and repeated measures data collection	N/A (the paper presented the methodology of the evaluation, but no results were presented or discussed)	SNS; Self-reported behavior change: +; Objectively measured behavior change: + ; Engagement: N/A
Gabarron et al, 2012 [67]	Sexual health / Young adults	Virtual Clinic for Sexually Transmitted Diseases (VCSTD) / Avatars	Impact evaluation: Methodology to examine usefulness of service / user experience through online feedback forms—behavior change through online questionnaires—usage data / effect of the interventions on (1) number of abortions, (2) number of chlamydia tests, (3) amount of emergency contraception information sold	N/A (presented the methodology of the evaluation, but not the results)	SNS; Acceptability/ user engagement: N/A Self-reported behavior change: N/A
Kelty et al, 2012 [68]	Physical activity/ teenage girls	Facebook / “Girls’ recreational activity support program using information technology”	RCT: evaluating a baseline intervention (based on face-to-face support) and an intervention based on Facebook pages; data collected during a 3-month period. Study aimed to evaluate the effectiveness of social networking intervention for improving physical activity and behavior change, as well as the feel of support to the users of the service	Although intervention group increased physical activity, the difference between the 2 interventions was not significant. Engagement with the online component was low. Additional strategies are required to improve engagement and compliance with social networking interventions based on Facebook	SNS; Adherence-engagement: +; Objectively measured behavior change (based on physiological data, BMI): ++++
Laakso et al, 2012 [69]	Self-management of chronic disease	HOFA (Healthy Outcomes for Australians): Social media platform for information sharing, community building, and social networking for those with chronic disease	Lit review: No evaluation of effectiveness. Lit review informed the design of the intervention. Paper presents the results of the review and a general description of the HOFA website	N/A (paper included a review of the relevant literature)	N/A
Hwang et al, 2012 [70]	Weight loss/ Adults	SparkPeople.com/ Discussion forum and blogs	Observational study: finding an association between frequency of use of social media & social support in the context of weight loss/ survey	Using social media tools of an online weight loss program at least 1x/wk is strongly associated with receiving encouragement, but not information or shared experiences	SNS; Self-reported behavior change: ++

^a^Abbreviations and symbols used in this column are explained in Multimedia Appendix 1.

^b^Conflict of interest declared.

**Table 2:** Role of theory and relationship between top-down and bottom-up features in interventions.^a^

Reference/ intervention name	Theories or models used (if any)	Role of top-down design (HP) in intervention	Role of bottom-up or emergent SNS features	Citizen-led or participatory elements (if any)	Relation between HP and SNS in study
An et al, 2008 [32]	N/A	Quitplan website	Observed usage patterns	N/A	^b^HP ←→ SNS
Baghaei et al, 2009 (SOFA) [33]	N/A	Educational content	Usage patterns	N/A	HP ←→ SNS
Burke & Oomen-Early, 2008 [34]	Bloom’s Taxonomy of Educational Objectives	High school teaching idea (guided use of SNS)	Learning from SNS expected	Students learn advocacy campaigning and citizen debates	HP → SNS
Cobb et al 2010 (QuitNet) [35]	Social Network Analysis	Design of smoking interventions	Social networking analysis results inform HP	N/A	HP ← SNS
Cunningham et al, 2008^c^ (Alcohol_HelpCenter) [36]	N/A	Expert forum moderator	Observed usage and content inform HP	N/A	HP ← SNS
Falan et al, 2011 (SCEDES) [37]	N/A	Nurses, educators in community	Planned bottom-up flow of knowledge	Planned consumer empowerment	HP ←→ SNS
Foster et al, 2010 (StepMatron) [38]	N/A	Design of intervention	Peer pressure	N/A	HP ←→ SNS
Fukuoka et al, 2011 [39]	N/A	Planned anti-diabetes intervention	Planned social support in community	Focus group emergent themes help determine intervention	HP ←→ SNS
Gasca et al, 2009 (pHealthNet) [17]	N/A	Design of intervention based on existing hospital support groups	Peer-to-peer challenges, games, experience sharing, community attachment	Researchers consulted support groups to determine technology design	HP ←→SNS
Gay et al, 2011 (AURORA) [40]	N/A	Design of intervention based on effects of emotional health on physical health	Visual emotion sharing (selecting Flickr pictures)	N/A	HP ←→ SNS
Kamal et al, 2010 [41]	Social Science Theories (U&G; CICB; SI; OC; SNT; DI) and Behavior Change Theories (TTM; HBM; SCT; TRA)	Intervention design based on survey of models and theories	Planned SNS should promote social belonging, identity and comparison (grounded in theories)	N/A	HP → SNS
Kharrazi et al, 2011 [42]	TPB	Educational materials + pedometer linked to personal health record	Planned SNS should enable peer pressure, competition, and rewards	Interactive personal health record should empower consumer	HP → SNS
Krukowski et al, 2008 [43]	N/A	Website design with educational content	Bulletin board, Web chats, stories, biographies	Focus groups help to determine website features	HP ←→ SNS
Lindsay et al, 2009 [44]	N/A	Moderator support	Online closed community	N/A	HP ←→ SNS
Linehan et al, 2010 [45]	N/A	Planned intervention for general nutrition education	Participants upload photos of meals to be tagged anonymously for nutrition value	Nutrition tagging generated by participants	HP → SNS
Liu & Chan, 2010 [46]	Social Support Theory; Social Identity Theory (SI); HBM	Virtual community management based on theories and evidence	Observed social support patterns in SNS inform interventions	N/A	HP ← SNS
Maibach et al, 2007 [47]	Ecological models: people-based and place-based fields of influence	Planned framework for Social Marketing to promote behavior change in SNS	Theory of SNS as people-based fields of influence	Participatory model considered	HP → SNS
Munson et al, 2010 (3GT) [48]	Positive Psychology	Encouraging sharing of positive events in SNS	Real attitudes of SNS users	N/A	HP ←→ SNS
Nahm et al, 2009 (TSW) [49]	Social Cognitive Theory	Theory-based website with moderated discussion	Emerging themes from discussion	N/A	HP ←→ SNS
Nordfelt et al, 2010 (Diabit) [19]	N/A	Educational materials on website	Attitudes from essays written by participants	Attitudes and suggestions provide input for further development of website	HP ← SNS
O’Grady et al, 2008 [50]	Kolb Model of Experiential Learning	Design of collaborative health education	Harnessing of SNS technology to support learning	Patients may be considered as authoritative due to their experience	HP → SNS
Olsen & Kraft, 2009 [51]	N/A	Future designs based on observed SNS features	Aspects of SNS perceived by users as promoting social support and adherence	Attitudes of SNS users provide input to technical design of SNS technology (positive and negative experiences/ concerns)	HP← SNS
Potente et al, 2011 [52]	N/A	Social Marketing use of social media	Sharing and debating video online (YouTube, Twitter, forums)	N/A	HP ←→ SNS
Rhodes et al, 2010 (CyBER/ M4M) [53]	Social Cognitive Theory (SCT); Grounded Theory used for data analysis	Chat room educators	Observed chat rooms interactions with educators inform intervention design	Methodology: Community-Based Participatory Research (CBPR)	HP ←→ SNS
Richardson et al, 2010 [54] (SUH)	SCT	SUH intervention	Observed community engagement and peer support	N/A	HP ←→ SNS
Roblin, 2011 [55]	Social support	Planned diabetes intervention	Peer-to-peer mobile messages	Participatory model for diabetes management	HP ←→ SNS
Stoddard et al, 2008 [56] (Smokefree.gov)	N/A	Smoking intervention	Observed bulletin board usage and effectiveness	N/A	HP ←→ SNS
Toscos et al, 2010 [57]	For qualitative analysis: Presentation of Self in Everyday Life & Cognitive Dissonance	Future designs based on SNS observations	Commonly mentioned barriers to PA in forum to inform HP design	N/A	HP ← SNS
Waters et al, 2011 [58]	Dialogic Theory	University Health Centers	Health Center SNSs’ use of Dialogic Principles	N/A	HP ←→SNS
West et al, 2011 [59]	Integrated Behavioral Model (IBM): to code constructs for behavioral support.	Health education on breastfeeding	Observed peer support via blogging to inform HP interventions	N/A	HP ← SNS
Woodruff et al, 2007 [60]	MI	MI used within virtual chat room	Peer pressure and social support	Participatory research involving schools and academics	HP ←→ SNS
Young et al, 2010 [61]	Persuasion Design Principles (PSD)	PA website with pedometer	Harness peer pressure using micro-blogging	Teenagers were consulted about design principles	HP → SNS
Kamal et al, 2013 [18]	ABC: A theoretical framework encompassing concepts from 13 individual theoretical models	Design & content components of a social networking tool were informed from the ABC theoretical framework	N/A (the study involved only a prototype)	Researchers involved users in the prototype design and evaluation phase	HP → SNS
Ploderer et al, 2013 [63]	N/A	Smoking cessation Facebook support group	Analysis of posts made to a Facebook support group by 180 users	Analysis of users’ posts	SNS → HP
Baelden et al, 2012 [62]	N/A	Design of the tool was based on participatory approaches	Observation of usage statistics following 3 implementation scenarios: (1) voluntary (with 15,000 users), (2) semi-voluntary (with 1431 users), & (3) curriculum integration (with 161 users). Each implementation phase lasted ~1 month	Researchers involved users in prototype design and evaluation phase (through focus group interviews)	HP← SNS
Gold et al, 2012 [64]	N/A	Design of intervention was based on collaboration between public health professionals, experts in user experience, and people from creative industries	Observation of usage statistics	N/A	HP ← SNS
Nguyen et al, 2013 [65]	Concept of edutainment	Design of tool was based on the concept of edutainment	Observation of usage statistics + online surveys	N/A	HP ← SNS
Kolt et al, 2013 [66]	N/A	N/A	Observation of participants self-reported behavior including data on physical activity levels, self-reported quality of life, user satisfaction, psychosocial correlates	N/A	SNS → HP
Gabarron et al, 2012 [67]	Gaming and eLearning approach	Design of tools involved an avatar, which was influenced by gaming and eLearning concepts	Feedback forms; online questionnaires and publicly available usage data	N/A	SNS → HP
Kelty et al, 2012 [68]	N/A	N/A	Objectively measured effect (eg, use of pedometers; digital scales, calculation of BMI and CRF scores)	N/A	SNS → HP
Laakso et al, 2012 [69]	N/A (based on lit review of the barriers to accessing and managing health information)	Interdisciplinary input from specialists in physiotherapy, exercise science, nutrition, education, human services, psychology	N/A	N/A	N/A
Hwang et al, 2012 [70]	N/A	N/A	Questionnaire survey, interviews, qualitative analysis of posts in discussion forums	N/A	SNS → HP

^a^PA: Physical activity; Social Science theories: U &G: Uses and Gratification theory [71], CICB: Common Identity and Common Bond theories [72], OT: Organizational Commitment theory [73], SI: Social Identity theory [74,75], SST: Social Support Theory [76,77], SNT: Social Network Threshold [78], DI: Diffusion of Innovation theory [79]; Behavior change theories: SCT: Social Cognitive Theory [28], TTM: Transtheoretical Model [80], TPB: Theory of Planned Behavior [27], TRA: Theory of Reasoned Action (see TPB), HBM: Health Belief Model [81], MI: Motivational Interviewing.

^b^The following notations have been used to denote the relationship between HP and SNS in the study: HP ←→ SNS (emphasis on top-down design); HP ← SNS (emphasis on bottom-up flow of knowledge through observation and/or participation); HP → SNS (both aspects included in the study).

^c^Conflict of interest declared.

### Effectiveness Studies

#### Overview

A total of 26 studies (Table 1) had an explicit focus on effectiveness. These were RCTs (n=6), fully powered and explicitly designed observational studies (n=5), and pilot studies (n=15). A total of 17 articles (Table 1) did not report results on the effectiveness of social networking for health promotion. The studies presented in these articles were either planned interventions, conceptual frameworks, and early prototypes—usually coupled with findings from a literature review [34,37,39,41,42,46,47,50,58,67,69] or showed results other than those related to the measurement of the effectiveness of social networking applications. For example, findings were focused on the information seeking and sharing behavior of users of social media, or the application of social network analysis to show the growth and characteristics of Web 2.0 applications [35,49,19,51,53,57]. The main findings of the 26 studies with a focus on effectiveness are summarized below.

#### Randomized Controlled Trials

Six studies were RCTs [44,54,56,60,66,68]. Of these, three studies [54,66,68] examined the effect of online social networking on objectively measured behavior, while the remaining studies attempted to examine this effect on self-reported behaviors. In the case of objectively measured behaviors, Kolt et al [66] presented the methodology, but not actual results from the study. Richardson et al [54] and Kelty et al [68] showed no significant effect on physical activity (in terms of walking behavior) between the baseline and online social networking interventions. However, the two studies showed mixed results in terms of the level of engagement and adherence with socially mediated interventions. Richardson et al [54] reported a positive effect of an online community on adherence (ie, engagement and completion of the intervention) while Kelty et al [68] showed a low level of engagement.

Researchers who examined self-reported behavior change using RCTs presented a mixed picture of online social networking versus behavior change in the context of smoking cessation, healthy eating, and physical activity. Stoddard et al [56] measured the effect of a bulletin board on smoking abstinence (n=1375, 50:50 allocation to bulletin board vs usual care)—only 11% in the intervention arm viewed or posted to the bulletin board, and no significant effect was found. Woodruff et al [60] found a short-term self-reported effect on smoking abstinence. However, the study evaluated the whole intervention (which included motivational interviewing) thus making it difficult to determine the effect of the social networking aspects. The effect of a specific HP component in a health care social network was evaluated by Lindsay et al [44], who studied the effect of removing a moderator from an online community. The 12-month study involved 108 coronary heart patients, half of whom were randomly assigned to Web portal access. For both groups, moderation was removed after 6 months. After 3 months of non-moderated usage, there was a significant reduction in self-reported healthy behaviors for both groups. During the moderated phase, there was a positive effect for the portal (intervention) group.

#### Observational Studies

Four studies determined effectiveness through controlled observational designs. An et al [32] found a weak association between community engagement and abstinence (smoking) using multivariate and path analyses. Krukowski et al [43] used exploratory factor analysis to determine which website features were associated with actual weight loss (n=123). “Social support” was the highest predictor. Similar findings were presented by Hwang et al [70]. The researchers found that using the social networking tools of an online weight loss website was strongly associated with receiving encouragement and support from the community. However, no strong associations were observed between the use of social networking tools and the amount of new information or shared experiences received. Ploderer et al [63] examined the relationship between stages of health identity change and seeking social support. They performed a quantitative analysis of messages posted in a public Facebook support group for smoking cessation. The findings showed that supportive responses and leadership came from users who had just started their behavior change process rather than people who successfully completed it. Finally, West et al [59] performed both qualitative and quantitative analyses of a large set of blog posts to determine whether blogging can promote breastfeeding. The findings showed that sharing personal experiences was more likely to elicit behavioral intention than generic advice or information.

#### Pilot Studies

A total of 14 articles examined the effectiveness of social networking interventions in studies that were pilots (with regard to the power to detect the effect of interest) or qualitative explorations. In the majority of cases, researchers recruited small sample sizes and employed mixed (qualitative and quantitative) methods. Typical data collection techniques were focus groups, online questionnaire surveys, interviews, and quantitative analysis of user-generated content (such as posts in blogs, discussion forums, and other social networking sites).

Nine studies [18,33,36,38,40,45,52,55,61] showed a positive effect of social networking interventions on engagement/acceptance of technology and behavior change. In particular, several studies [18,33,45] showed that social networking interventions enhanced user engagement and acceptance of technology in the contexts of obesity, healthy eating, and physical activity. Similar findings were reported in the case of interventions related to alcohol misuse and diabetes [36,38]. In addition to positive user engagement, two studies [38,61] demonstrated promotion of walking (gradual increase in the number of steps). Positive behavior changes were self-reported [40,52]. Gay et al [40] focused on the application of social networking in the context of emotional health. The results were positive for emotion awareness, sharing, and social support. Finally, Potente et al [52] showed a high level of self-reported risk awareness in the context of sun protection.

The remaining five studies [17,48,62,64,65] presented mixed results regarding the effectiveness of social networking interventions in health promotion. Several studies [62,64,65] were focused on sexual health promotion (including HIV protection). The findings of these studies showed that social networking can be a useful tool for initiating online discussions. However, several limitations were identified, such as low level of participation and engagement on a voluntary basis, lack of expected “viral” recruitment through online networks, and problems maintaining user engagement in the long term. In addition to sexual health, two studies [17,48] that were focused on obesity and emotional health reported similarly mixed effectiveness. In particular, Gasca et al [17] showed a high level of acceptance of technology, but the authors reported also that social networking did not support long-term behavior change (ie, low sustainability of behavior change). In Munson et al [48], the positive engagement with technology was counteracted by concerns about privacy and personal information management.

### Theoretical Grounding

Twenty studies involved interventions that were grounded in social and psychological theories, or technological model and approaches. Most of these were early stage designs that we classed as top-down studies in Table 2. Many were based on the expected emergent properties of social networks. In particular, Kamal et al 2010 [41] grounded their intervention design on a survey of theories relating to social networking and behavior change. The social networking theories employed were Uses and Gratification (U&G) theory [71]: participants use media actively and search for specific resources (for usefulness or gratification); Common Identity and Common Bond (CICB) theories [72]: online communities need to be managed in a way that facilitates attachment to a group (Common Identity) and attachment to group members (Common Bond) in order to sustain voluntary participation; Organizational Commitment theory (OT) [73]: a model of different kinds of commitment (or attachment) to an organization, which can be relevant to an online community; Social Identity (SI) theory [74,75]: motivation for behavior change is influenced by the sense of belonging to a group; Social Support Theory (SST) [76,77]: in social networks, social support might take the form of messages showing empathy, encouragement and caring (among others), which may be beneficial for health and positive mental attitude, including motivation for behavior change; Social Network Threshold (SNT) [78]: this theory distinguishes critical/threshold numbers of individuals’ contacts influencing their adoption behavior from the effects of structural aspects regarding individuals’ positions in social networks; and Diffusion of Innovation (DI) theory [79]: populations comprise a theoretical distribution of people with different propensities for adopting innovations, from “innovators” and their “early adopters” to “laggards”.

The planned social network should promote a sense of belonging and social identity (based on SI and CICB theories) as well as social support (based on SST) among other features. Social support theory was also applied in other interventions [46,55]. In a follow-up paper, Kamal et al [18] summarized the individual theoretical models into the ABC framework*.* This informed the design of the VivoSpace, a social networking tool focused on healthy living.

Other theories used were as follows: People-based and Place-based fields of influence, where people are influenced by the places they are in, as well as other people (norms, etc) [47]; Positive psychology [82], used by Munson et al 2010 [48] (3GT), in which sharing of positive stories and experiences promotes emotional health (acceptance evaluation); Social Cognitive Theory used for the whole intervention design in three studies with moderated discussion [49,53,54]; Theory of Planned Behavior, in which peer-pressure (norms) should emerge in planned social network for sharing step count data [42]; Kolb Model of Experiential Learning [83], in which learning happens through experience, and experience sharing [50]; Dialogic Theory [84] used in one study [58] to evaluate university health center use of Facebook; Motivational Interviewing (MI) used for chat room educators [60]; and Persuasion Design Principles (PSD) used for website design [61].

A few studies were not theoretically grounded but instead based on commonly held expectations about the effects of social networking. For example, AURORA [40] was focused on the expected positive effects on emotional health if positive experiences are shared. However, this can also be negative, due to contagion of negative emotions. Another was Tagliatelle [45], which is based on the expectation of constructive social tagging of meals. Nguyen et al [65] designed an intervention for sexual education using Facebook. The intervention followed the concept of edutainment to support adherence and engagement. Finally, the Virtual Clinic for Sexually Transmitted Diseases [67] was an Avatar-supported intervention, the design of which was based on concepts from gaming and eLearning to support adherence and promote behavior change among the users of the service.

## Discussion

### Principal Considerations

The aim of this study was to review the existing evidence about the effectiveness of SNS in health promotion. As opposed to existing systematic reviews, this study took a different approach by including a broader range of studies for review. The selected papers reflected different dimensions of effectiveness and types of a research design. This decision was made in order to address some of the gaps identified in previous reviews of the relevant literature, and in particular, the focus on RCTs (ignoring other types of research designs), as well as the narrow focus of effectiveness on behavior change (excluding other types of effectiveness that may have an impact on our understanding of behavior change, like usability, user satisfaction, level of adherence, and technology acceptance). By reviewing a larger pool of papers in this context, our objectives were to extend our existing knowledge about how effectiveness is being measured and identify the level of uptake of theories in the design of interventions based on online social networking.

### Effectiveness of Social Networking Sites

In accordance with findings from previous reviews [1,2], the RCTs included in this review showed no clear effect of SNS on objectively measured behavior change (eg, no significant increase in walking behavior in the context of obesity-related interventions [54,68]). However, more positive effects on both self-reported and objectively measured behavior change were reported in the case of small pilot studies [38,61]. It is well recognized that small pilot studies often show a more promising positive effect of an intervention than later larger and more pragmatic evaluations [85].

The review of controlled observational studies showed some interesting aspects about the role of social support in behavior change. It appears that not all aspects of SNS (eg, social support, peer pressure, or information sharing) have an equal role. In particular, social support was the highest predictor of behavior change in the context of weight loss [43]. Also, the use of SNS in weight loss interventions was more strongly associated with receiving encouragement and support from the community rather than the amount of new information and experiences received [70]. Finally, there was evidence that social support is not manifested equally among members of an online community. The level of completion of behavior change appeared to be an important predictor of social support, with users who had just started their behavior change being more supportive than their peers who successfully completed it [63]. In previous reviews of the literature [1,2], social support was identified as a positive aspect of interventions delivered through SNS. However, this review goes a step further by highlighting its role in relation to other aspects of SNS, like peer pressure and information sharing, but also among different members of the online community. Future research should investigate in more depth the role of social support as a specific component of health promotion interventions and for interface design. For example, what is the effect of different contextual factors on online social support? Or how can the interface design of SNS applications be enhanced with features that could motivate social support among different members of the online community?

Broader influences on effectiveness, such as usability or level of engagement, were reported more frequently in pilot studies, rather than RCTs and observational research. The majority of pilot studies showed results about the level of engagement with an online social networking application over a short period of time (normally between 1-4 weeks). Despite the fact that all authors reported systematically a good level of engagement at the beginning of the trial period, in many cases the number of active users dropped considerably in the long term [17,48,62,64,65]. Only a few authors attempted to explain the reasons for this phenomenon. However, when this information was reported, the most common reasons included concerns about privacy, problems related to personal information management, and lack of motivation [48,53]. Only in one pilot study did the authors examine what actions should be taken to improve the level of adherence and engagement with SNS [51]. They found that dynamic and interactive elements (such as online games and contests) could improve adherence. The lack of active participation and long-term engagement with SNS technology was an issue also in the case of RCTs. For example, Stoddard et al [56] reported that only 11% of participants were active users (ie, posted or viewed comments/messages), while Woodruffe et al [60] found a significant self-reported behavior change only in the short term. A reduction in the level of engagement in RCTs has been reported by other authors as well [2,86,87]. Also, it is interesting that almost all RCTs in our review, except for one, did not exceed a 12-week trial period. This shows a lack of evidence about the level of user engagement and retention in the case of longer trial periods (such as 12 months or more). The lack of long-term RCTs (ie, more than a year) is a typical phenomenon in this context and similar concerns regarding long-term user engagement and retention have been expressed by other authors in the past [2].

Lack of clear evidence was evident in the case of the evaluation of the usability and technology acceptance of the SNS. Despite the fact that usability was frequently mentioned in several papers as a feature of a well-designed social networking application, there was no evidence of complete usability tests or heuristic evaluations. In the majority of cases, usability was reduced to the evaluation of the quality of the contents and information in an SNS [58]. In other cases, some authors reported the application of a participatory design approach to inform the development of usable interfaces for SNS. This was more common in interventions with a health-focused SNS component rather than the mainstream SNS channels, like Facebook. Evaluating the usability (ie, interface design) of SNS applications is important for both user engagement and behavior change [88]. Also, this type of evaluation will provide some of the evidence needed by informaticians to design ease-of-use SNS interfaces for health promotion interventions. Finally, the review showed a lack of studies examining technology acceptance (ie, studies focused on identifying and modeling factors of technology acceptance and intention to use the specific technology).

### Use of Theory in the Design of Social Networking Sites

As opposed to previous reviews of the literature [2], the papers included in this review showed a wider range of social and behavioral theories and design approaches used to inform the design of interventions. This finding shows that more researchers are choosing a more theory-driven approach as a means of achieving powerful effects [11]. Although a wide range of theories were mentioned in the studies, the social networking concepts that they emphasized were often overlapping. The most common were peer pressure, social support, and sense of identify (ie, belonging to a community).

Of the 20 papers that showed evidence about the use of theory to inform the design of interventions, the authors evaluated effectiveness in only half. In the context of physical activity, smoking cessation, and diabetes, the findings showed a positive effect of interventions grounded on persuasion design [61], motivational interviewing [60], and social support theory [55] on behavior change (both self-reported and objectively measured) respectively. Also, interventions based on the ABC framework [18] and positive psychology [48] showed good level of engagement and a positive effect on behavioral intention to share personal experiences. Positive, but not statistically significant, effect on behavior change was reported by authors who applied social cognitive theory to the design of an SNS intervention for physical activity [54], while the results were mixed in terms of engagement in the case of an SNS intervention grounded on the concept of edutainment [65].

Theories were used a priori to inform the design and contents of the online intervention. However, in the majority of cases, authors were not clear as to which aspects of the theory were applied specifically for the delivery of the SNS. This was common for interventions encompassing a website, part of which was the social networking application. In a few cases, the researchers also used bottom-up approaches to enhance the design and the contents of SNS. Bottom-up approaches were based on the use of observation using information extraction tools and social network analysis [49,59,57,35]. Yet, no study showed clearly how both top-down and bottom-up approaches to the design of health promotion interventions can be integrated into an iterative design life-cycle or how top-down design of health promotion can be linked with bottom-up observation and user participation.

### Limitations

This review has several limitations. Only articles indexed in Google Scholar or PubMed were included. However, most academic publications are found by Google Scholar. We did not include gray literature such as white papers and unpublished reports. In addition, our search terms may have missed some relevant articles, especially in the context of health prevention and preventive strategies. However, health prevention was not the focus of this review and a decision was made to include in the search for relevant papers only terms representing prevention strategies that are known examples of integrative (mixed) health promotion interventions, that is, health promotion interventions that incorporate methods from prevention strategies, like social marketing and health education. Finally, due to the exploratory nature of this review, we decided to include a range of study designs, at various stages of completeness. This made it difficult to assess the risk of bias or perform a meta-analysis of the papers included in the analysis. Therefore, the findings should be interpreted with caution.

### Conclusions

Narrative approaches to evidence synthesis that incorporate diverse literature can be valuable in highlighting issues beyond simple summary measures of effect. Indeed, a simple meta-analysis of this evidence base would be misleading given the heterogeneity of the interventions. Instead, this review has identified theoretical and empirical issues related to the success of health promoting interventions that harness social media. We have shown that more, and longer, RCTs need to be conducted that take into account contextual factors such as patient characteristics and types of SNS. Also, more evidence is needed regarding the actual usability of SNS and how different interface design elements may help or hinder behavior change and engagement. It will be crucial to investigate further the effect of theory on the effectiveness of SNS for health promotion. The informatics research in this field needs better designed experiments. Public health practitioners need to prepare for more action research whereby theoretically founded interventions generate evidence that helps them to evolve—reflecting the emergent nature of social technologies.

## Appendix

Multimedia Appendix 1 List of extracted items.

## Abbreviations

CICB: Common Identity and Common Bond theories
DI: Diffusion of Innovation theory
HBM: Health Belief Model
HP: health promotion
MI: Motivational Interviewing
OT: Organizational Commitment theory
PA: physical activity
RCT: randomized controlled trials
SCT: Social Cognitive Theory
SI: Social Identity theory
SNS: social networking sites
SNT: Social Network Threshold
SST: Social Support Theory
TPB: Theory of Planned Behavior
TRA: Theory of Reasoned Action
TTM: Transtheoretical Model
U&G: Uses and Gratification theory

## References

[1] Maher C, Lewis Lucy K, Ferrar Katia, Marshall Simon, De Bourdeaudhuij Ilse, Vandelanotte Corneel (2014). Are health behavior change interventions that use online social networks effective? A systematic review. J Med Internet Res.

[2] Laranjo Liliana, Arguel Amaël, Neves Ana L, Gallagher Aideen M, Kaplan Ruth, Mortimer Nathan, Mendes Guilherme A, Lau Annie Y S (2015). The influence of social networking sites on health behavior change: a systematic review and meta-analysis. J Am Med Inform Assoc.

[3] Valente T W (2010). Social networks and health: models, methods and applications.

[4] Wicks Paul, Massagli Michael, Frost Jeana, Brownstein Catherine, Okun Sally, Vaughan Timothy, Bradley Richard, Heywood James (2010). Sharing health data for better outcomes on PatientsLikeMe. J Med Internet Res.

[5] Rozenblum Ronen, Bates David W (2013). Patient-centred healthcare, social media and the internet: the perfect storm?. BMJ Qual Saf.

[6] Hamm Michele P, Chisholm Annabritt, Shulhan Jocelyn, Milne Andrea, Scott Shannon D, Klassen Terry P, Hartling Lisa (2013). Social media use by health care professionals and trainees: a scoping review. Acad Med.

[7] Korda Holly, Itani Zena (2013). Harnessing social media for health promotion and behavior change. Health Promot Pract.

[8] Eysenbach Gunther, Powell John, Englesakis Marina, Rizo Carlos, Stern Anita (2004). Health related virtual communities and electronic support groups: systematic review of the effects of online peer to peer interactions. BMJ.

[9] Chang Tammy, Chopra Vineet, Zhang Catherine, Woolford Susan J (2013). The role of social media in online weight management: systematic review. J Med Internet Res.

[10] Schein R, Wilson K, Keelan JE (2011). Peel Public Health Report.

[11] Webb Thomas L, Joseph Judith, Yardley Lucy, Michie Susan (2010). Using the internet to promote health behavior change: a systematic review and meta-analysis of the impact of theoretical basis, use of behavior change techniques, and mode of delivery on efficacy. J Med Internet Res.

[12] Van De Belt Tom H, Engelen Lucien J L P G, Berben Sivera A A, Schoonhoven Lisette (2010). Definition of Health 2.0 and Medicine 2.0: a systematic review. J Med Internet Res.

[13] Syed-Abdul Shabbir, Fernandez-Luque Luis, Jian Wen-Shan, Li Yu-Chuan, Crain Steven, Hsu Min-Huei, Wang Yao-Chin, Khandregzen Dorjsuren, Chuluunbaatar Enkhzaya, Nguyen Phung Anh, Liou Der-Ming (2013). Misleading health-related information promoted through video-based social media: anorexia on YouTube. J Med Internet Res.

[14] Chou W, Hunt Yvonne, Folkers Anna, Augustson Erik (2011). Cancer survivorship in the age of YouTube and social media: a narrative analysis. J Med Internet Res.

[15] Pedrana A, Hellard Margaret, Gold Judy, Ata Nadine, Chang Shanton, Howard Steve, Asselin Jason, Ilic Olivia, Batrouney Colin, Stoove Mark (2013). Queer as F**k: reaching and engaging gay men in sexual health promotion through social networking sites. J Med Internet Res.

[16] Mackert M, Kim Eunice, Guadagmo Marie, Donovan-Kicken Erin (2012). Using Twitter for prenatal health promotion: encouraging a multivitamin habit among college-aged females. Stud Health Technol Inform.

[17] Gasca E, Favela J, Tentori M (2009). Assisting Support Groups of Patients with Chronic Diseases through Persuasive Computing. Journal of Universal Computer Science.

[18] Kamal N, Fels S, Blackstock M, Ho K (2013). The ABCs of designing social networksfor health behaviour change: the Vivospace social network. Advances in network analysis.

[19] Nordfeldt Sam, Hanberger Lena, Berterö Carina (2010). Patient and parent views on a Web 2.0 Diabetes Portal--the management tool, the generator, and the gatekeeper: qualitative study. J Med Internet Res.

[20] Eysenbach Gunther (2009). Infodemiology and infoveillance: framework for an emerging set of public health informatics methods to analyze search, communication and publication behavior on the Internet. J Med Internet Res.

[21] Evans D (2006). How social marketing works in healthcare. BMJ.

[22] Griffiths J (2008). Social marketing for health and specialised health promotion.

[23] World Health Organization Health Education: theoretical concepts, effective strategies and care competences.

[24] Nielsen J (2012). Usability 101: introduction to usability.

[25] Nielsen J (2012). User satisfaction vs performance metrics.

[26] Venkatesh V, Bala H (2008). Technology Acceptance Model 3 and a Research Agenda on Interventions. Decision Sciences.

[27] Ajzen I (1991). The Theory of Planned Behaviour. Organizational Behavior and Human Decision Processes.

[28] Bandura A (1986). Social foundations of thought and action: a social cognitive theory.

[29] Rodgers M, Sowden A, Petticrew M, Arai L, Roberts H, Britten N, Popay J (2009). Testing Methodological Guidance on the Conduct of Narrative Synthesis in Systematic Reviews: Effectiveness of Interventions to Promote Smoke Alarm Ownership and Function. Evaluation.

[30] Howland Jl, Wright TC, Boughan RA, Roberts BC (2009). How Scholarly Is Google Scholar? A Comparison to Library Databases. College & Research Libraries.

[31] Walters WH (2007). Google Scholar coverage of a multidisciplinary field. Information Processing & Management.

[32] An Lawrence C, Schillo Barbara A, Saul Jessie E, Wendling Ann H, Klatt Colleen M, Berg Carla J, Ahulwalia Jasjit S, Kavanaugh Annette M, Christenson Matthew, Luxenberg Michael G (2008). Utilization of smoking cessation informational, interactive, and online community resources as predictors of abstinence: cohort study. J Med Internet Res.

[33] Baghaei N, Freyne J, Kimani S, Smith G, Berkovsky S, Bhandari D, Colineau N, Paris C (2009). SOFA: an Online Social Network for Engaging and Motivating Families to Adopt a Healthy Lifestyle. OZCHI 09.

[34] Burke S, Oomen-Early J (2008). That’s Blog Worthy. American Journal of Health Education.

[35] Cobb Nathan K, Graham Amanda L, Abrams David B (2010). Social network structure of a large online community for smoking cessation. Am J Public Health.

[36] Cunningham John A, van Mierlo Trevor, Fournier Rachel (2008). An online support group for problem drinkers: AlcoholHelpCenter.net. Patient Educ Couns.

[37] Falan S, Han B, Rea A (2011). A Smart Consumer-empowered Diabetes Education System (SCEDES): Integrating Human Wellbeing and Health Care in the Community Environment. AMCIS 2011 Proceedings.

[38] Foster D, Linehan C, Kirman B, Lawson S, James G MindTrek 2010.

[39] Fukuoka Yoshimi, Kamitani Emiko, Bonnet Kemberlee, Lindgren Teri (2011). Real-time social support through a mobile virtual community to improve healthy behavior in overweight and sedentary adults: a focus group analysis. J Med Internet Res.

[40] Gay Geri, Pollak Jp, Adams Phil, Leonard John P (2011). Pilot study of Aurora, a social, mobile-phone-based emotion sharing and recording system. J Diabetes Sci Technol.

[41] Kamal N, Fels S, Ho K (2010). Online Social Networks for Personal Informatics to Promote Positive Health Behavior.

[42] Kharrazi H, Vincz L, Stephanidis C, Stephanidis C (2011). Increasing Physical Activity by Implementing a Behavioral Change Intervention Using Pervasive Personal Health Record System: An Exploratory Study. Universal Access in Human-Computer Interaction: Applications and Services.

[43] Krukowski Rebecca A, Harvey-Berino Jean, Ashikaga Takamaru, Thomas Colleen S, Micco Nicci (2008). Internet-based weight control: the relationship between web features and weight loss. Telemed J E Health.

[44] Lindsay Sally, Smith Simon, Bellaby Paul, Baker Rose (2009). The health impact of an online heart disease support group: a comparison of moderated versus unmoderated support. Health Educ Res.

[45] Linehan C, Doughty M, Lawson S, Kirman B, Olivier P, Moynihan P (2010). Tagiatelle: Social Tagging to Encourage Healthier Eating. CHI EA.

[46] Liu N, Chan H (2010). Understanding the Influence of Social Identity on Social Support Seeking Behaviors in Virtual Healthcare Communities. ICIS 2010.

[47] Maibach Edward W, Abroms Lorien C, Marosits Mark (2007). Communication and marketing as tools to cultivate the public's health: a proposed "people and places" framework. BMC Public Health.

[48] Munson SA, Lauterbach D, Newman MW, Resnick P (2010). Happier Together: Integrating a Wellness Application Into a Social Network Site.

[49] Nahm Eun-Shim, Resnick B, DeGrezia M, Brotemarkle R (2009). Use of discussion boards in a theory-based health web site for older adults. Nurs Res.

[50] O'Grady Laura A, Witteman Holly, Wathen C Nadine (2008). The experiential health information processing model: supporting collaborative web-based patient education. BMC Med Inform Decis Mak.

[51] Olsen E, Kraft P (2009). ePsychology: a pilot study on how to enhance social support and adherence in digital interventions by characteristics from social networking sites. Proceedings of the 4th International Conference on Persuasive Technology.

[52] Potente S, McIver J, Anderson C, Coppa K (2011). “It's a Beautiful Day … for Cancer”: An Innovative Communication Strategy to Engage Youth in Skin Cancer Prevention. Social Marketing Quarterly.

[53] Rhodes Scott D, Hergenrather Kenneth C, Duncan Jesse, Vissman Aaron T, Miller Cindy, Wilkin Aimee M, Stowers Jason, Eng Eugenia (2010). A pilot intervention utilizing Internet chat rooms to prevent HIV risk behaviors among men who have sex with men. Public Health Rep.

[54] Richardson Caroline R, Buis Lorraine R, Janney Adrienne W, Goodrich David E, Sen Ananda, Hess Michael L, Mehari Kathleen S, Fortlage Laurie A, Resnick Paul J, Zikmund-Fisher Brian J, Strecher Victor J, Piette John D (2010). An online community improves adherence in an internet-mediated walking program. Part 1: results of a randomized controlled trial. J Med Internet Res.

[55] Roblin Douglas W (2011). The potential of cellular technology to mediate social networks for support of chronic disease self-management. J Health Commun.

[56] Stoddard Jacqueline L, Augustson Erik M, Moser Richard P (2008). Effect of adding a virtual community (bulletin board) to smokefree.gov: randomized controlled trial. J Med Internet Res.

[57] Toscos T, Consolvo S, McDonald DW (2010). is it normal to be this sore?: using an online forum to investigate barriers to physical activity. Proceedings of the First ACM International Health Informatics Symposium.

[58] Waters RD, Canfield R, Foster JM, Hardy E (2011). Applying the dialogic theory to social networking sites. Journal of Social Marketing.

[59] West J, Hall PC, Hanson C, Thackeray R, Barnes M, Neiger B, McIntyre E (2011). Breastfeeding and Blogging. American Journal of Health Education.

[60] Woodruff Susan I, Conway Terry L, Edwards Christine C, Elliott Sean P, Crittenden Jim (2007). Evaluation of an Internet virtual world chat room for adolescent smoking cessation. Addict Behav.

[61] Young M (2010). Twitter Me: Using Micro-blogging to Motivate Teenagers to Exercise. Global Perspectives on Design Science Research -.

[62] Baelden D, Van Audenhove L, Vergnani T (2012). Using new technologies for stimulating interpersonal communication on HIV and AIDS. Telematics and Informatics.

[63] Ploderer B, Smith W, Howard S, Pearce J, Borland R (2013). Patterns of support in an online community for smoking cessation. Proceedings of the 6th international conference on communities and technologies.

[64] Gold Judy, Pedrana Alisa E, Stoove Mark A, Chang Shanton, Howard Steve, Asselin Jason, Ilic Olivia, Batrouney Colin, Hellard Margaret E (2012). Developing health promotion interventions on social networking sites: recommendations from The FaceSpace Project. J Med Internet Res.

[65] Nguyen Phuong, Gold Judy, Pedrana Alisa, Chang Shanton, Howard Steve, Ilic Olivia, Hellard Margaret, Stoove Mark (2013). Sexual health promotion on social networking sites: a process evaluation of The FaceSpace Project. J Adolesc Health.

[66] Kolt Gregory S, Rosenkranz Richard R, Savage Trevor N, Maeder Anthony J, Vandelanotte Corneel, Duncan Mitch J, Caperchione Cristina M, Tague Rhys, Hooker Cindy, Mummery W Kerry (2013). WALK 2.0 - using Web 2.0 applications to promote health-related physical activity: a randomised controlled trial protocol. BMC Public Health.

[67] Gabarron E, Serrano JA, Wynn R, Armayones M (2012). Avatars using computer/smartphone mediated communication and social networking in prevention of sexually transmitted diseases among North-Norwegian youngsters. Medical Informatics and Decision Making.

[68] Kelty T, Morgan P, Lubans D (2012). Efficacy and feasibility of the "Girls Recreational Activity Support Program Using Information Technology": a pilot randomised controlled trial. Advances in Physical Education.

[69] Laakso E, Armstrong K, Usher W (2011). Cyber-management of people with chronic disease: A potential solution to eHealth challenges. Health Education Journal.

[70] Hwang Kevin O, Etchegaray Jason M, Sciamanna Christopher N, Bernstam Elmer V, Thomas Eric J (2014). Structural social support predicts functional social support in an online weight loss programme. Health Expect.

[71] Ruggiero Te (2000). Uses and Gratifications Theory in the 21st Century. Mass Communication and Society.

[72] Yuqing Ren, Kraut R, Kiesler S (2007). Applying Common Identity and Bond Theory to Design of Online Communities. Organization Studies.

[73] Allen NJ, Meyer JP (1990). The Measurement and Antecedents of Affective, Continuance and Normative Commitment to the Organization J Occup Psychol, (63). Journal of Occupational Psychology.

[74] Tajfel H (1974). Social identity and intergroup behaviour. Social Science Information.

[75] Tajfel H, Turner JC (1986). The Social Identity Theory of Intergroup Behavior. The Social Psychology of Intergroup Relations.

[76] Cobb S (1976). Social support as a moderator of life stress. Psychosomatic Medicine.

[77] Minkler M (1981). Applications of Social Support Theory to Health Education: Implications for Work with the Elderly. Health Education Behavior.

[78] Valente TW (1996). Social network thresholds in the diffusion of innovations. Social Networks.

[79] Rogers EM (1983). Diffusion of innovation.

[80] Prochaska J O, Velicer W F (1997). The transtheoretical model of health behavior change. Am J Health Promot.

[81] Rosenstock IM, Strecher VJ, Becker MH (1988). Social Learning Theory and the Health Belief Model. Health Education & Behavior.

[82] Seligman Martin E P, Steen Tracy A, Park Nansook, Peterson Christopher (2005). Positive psychology progress: empirical validation of interventions. Am Psychol.

[83] Kolb DA (1984). Experiential learning: experience as the source of learning development.

[84] Kent ML, Taylor M (1998). Building dialogic relationships through the world wide web. Public Relations Review.

[85] Ioannidis JPA (2005). Why most published research findings are false?. PloS Med.

[86] Cavallo David N, Tate Deborah F, Ries Amy V, Brown Jane D, DeVellis Robert F, Ammerman Alice S (2012). A social media-based physical activity intervention: a randomized controlled trial. Am J Prev Med.

[87] Turner-McGrievy Gabrielle, Tate Deborah (2011). Tweets, Apps, and Pods: Results of the 6-month Mobile Pounds Off Digitally (Mobile POD) randomized weight-loss intervention among adults. J Med Internet Res.

[88] Caperchione Cristina M, Kolt Gregory S, Savage Trevor N, Rosenkranz Richard R, Maeder Anthony J, Vandelanotte Corneel, Duncan Mitch J, Van Itallie Anetta, Tague Rhys, Mummery W Kerry (2014). WALK 2.0: examining the effectiveness of Web 2.0 features to increase physical activity in a 'real world' setting: an ecological trial. BMJ Open.

