# Supplementary material for: The Role of Social Network Technologies in Online Health Promotion: A Narrative Review of Theoretical and Empirical Factors Influencing Intervention Effectiveness
Source: J Med Internet Res. 2015 Jun 11;17(6):e141. doi: 10.2196/jmir.3662 (PMC4526933; doi:10.2196/jmir.3662)
Supplement: Multimedia Appendix 1 [file jmir_v17i6e141_app1.pdf]

1. Multimedia Appendix 1. List of extracted items.

Effectiveness Studies

To identify the limits of current effectiveness evaluations of social networking in a health promotion intervention, we define the following sub-categories:

1. What is being evaluated?
    1. Whole intervention: HP + SNS together.
    2. HP component only (e.g. effect of moderator/educator in a forum).
    3. SNS component only (e.g. effect of online community within an HP intervention).

2. What kind of effectiveness?
    1. Usability and User Satisfaction .
    2. Adherence/Engagement and technology acceptance .
    3. Self-reported behaviour change.
    4. Objectively measured behaviour change (e.g. step counts).

3. Long-term or short-term?
    1. Short-term: during or shortly after an intervention (e.g. within 6 months).
    2. Long-term: sustained adherence or behaviour change (e.g. more than 6 months after start of an intervention).

In particular, in Table 1, we use the following notation in the column for effectiveness evaluation (if included in the study):

<1: component being evaluated>; <2: effect on what?>; <3: approximate level of evidence>

Field 1 can take one of three values: social networking component only (SNS), health promotion component only (HP), or whole intervention (SNS+HP). Field 2 is labelled according to the behaviour change required (e.g. smoking abstinence or treatment adherence), level of engagement, technology acceptance, user satisfaction and usability. Field 3 indicates the approximate level of evidence using the categories below:

RCT: objectively measured effect, long-term:	+++++
RCT: objectively measured effect, short-term:	++++
RCT: self-reported effect, long-term:	+++
RCT: self-reported effect, short-term:	++
Observational study: strong association:	++
Observational study: weak association:	+
Qualitative or pilot study: positive:	+
Qualitative or pilot study: mixed result:	+/-
RCT with no significant result:	0
Observational study with no clear associations:	0

Theoretical Grounding

In the case of theoretical grounding (Table 2) the following type of information was extracted after full-text review of the selected papers:

- Type of theory or model used (if any);

- Relationship between top-down and bottom-up approaches to intervention design. Theories, if any, that drive the top-down process were recorded, as well as any participatory process where citizens knowingly contributed bottom-up knowledge using social networking or other form of input;
- Relationship between health promotion and social network. In this case the following notation was used:
  - HP → SNS: Emphasis on top-down design
  - HP ← SNS: Emphasis on bottom-up flow of knowledge through observation and/or participation
  - HP ↔ SNS: Both aspects included in study.
